# Cardiometabolic multimorbidity and the risk of sudden cardiac death among geriatric community dwellers using longitudinal EHR-derived data

**DOI:** 10.3389/fendo.2025.1515495

**Published:** 2025-04-25

**Authors:** Yue Li, Zihan Mei, Zhengkun Liu, Ji Li, Guolei Sun, Marcus Eng Hock Ong, Jiancheng Chen, Haojun Fan, Chunxia Cao

**Affiliations:** ^1^ School of Disaster and Emergency Medicine, Tianjin University, Tianjin, China; ^2^ College of Management and Economics, Tianjin University, Tianjin, China; ^3^ Jinnan Hospital, Tianjin University, Tianjin, China; ^4^ Department of Emergency Medicine, Singapore General Hospital, Singapore, Singapore; ^5^ Health Services and Systems Research, Duke-NUS Medical School, Singapore, Singapore; ^6^ Xiamen Peiyang BCI & Smart Health Innovation Research Institution, Xiamen, China

**Keywords:** cardiometabolic multimorbidity, sudden cardiac death, mortality, older adult, electronic health records

## Abstract

**Background:**

Cardiometabolic multimorbidity (CMM) has increased globally in recent years, especially among geriatric community dwellers. However, it is currently unclear how SCD risk is impacted by CMM in older adults. This study aimed to examine the associations between CMM and SCD among geriatric community dwellers in a province of China.

**Methods:**

This study was a retrospective, population-based cohort design based on electronic health records (EHRs) of geriatric community dwellers (≥65 years old) in four towns of Tianjin, China. 55,130 older adults were included in our study. Older adults were categorized into different CMM patterns according to the cardiometabolic disease (CMD) status at baseline. The count of CMDs was also entered as a continuous variable to examine the potential additive effect of CMM on SCD. Cox proportional hazard models were used to evaluate associations between CMM and SCD. The results are expressed as hazard ratios (HRs) and 95% confidence intervals (CIs).

**Results:**

The prevalence of CMM was approximately 25.3% in geriatric community dwellers. Among participants with CMM, hypertension and diabetes was the most prevalent combination (9,379, 17.0%). The highest crude mortality rates for SCD were 7.5 (2.9, 19.1) per 1000 person-years in older adults with hypertension, coronary heart disease, diabetes and stroke (HR, 4.496; 95% CI, 1.696, 11.917), followed by those with hypertension, coronary heart disease, and stroke (HR, 3.290; 95% CI, 1.056, 10.255). The risks of SCD were significantly increased with increasing numbers of CMDs (HR, 1.787; 95% CI, 1.606, 1.987). The demographic, risk factors, serum measures and ECG-adjusted HR for SCD was 1.488 (1.327, 1.668) for geriatric community dwellers with an increasing number of CMDs.

**Conclusion:**

The risk of SCD varied by the pattern of CMM, and increased with increasing number of CMM among geriatric community dwellers.

## 1 Introduction

### 1.1 Background

Multimorbidity has become more prevalent across the globe in recent years, mainly for cardiometabolic conditions (1, 2). Cardiometabolic multimorbidity (CMM), the coexistence of two or more cardiometabolic diseases (CMDs), is related to higher disability, lower quality of life and increased health care costs (3, 4). A recent increase in life expectancy has resulted in a higher likelihood of individuals with single CMDs developing other CMDs, which has led to an increase in the prevalence of CMM (5). It is an emerging research area for public health, to understand the impact of CMM on the community (6). There is substantial evidence that CMM affects an estimated 30% of older adults (7). Over the past few years, the proportion of older adults in China has increased in China (8). The risk of cardiovascular death in older adults with CMM increases significantly with CMM (9) and CMM has become an increasingly challenging issue.

It is estimated that approximately half of all cardiovascular deaths are caused by sudden cardiac death (SCD), resulting in over 4-5 million deaths worldwide each year (10). SCD refers to an unexpected death or arrest from a cardiovascular cause (11). SCD is often fatal due to the short time available for effective medical intervention. Considering the poor prognosis of SCD, risk factors for progression to SCD in geriatric community dwellers are of concern. Several studies have demonstrated that any one of these CMDs alone may increase the risk of SCD (12, 13). However, previous studies on the association between CMDs and SCD have focused primarily on the association between a single disease and SCD, with little attention being given to the association between specific CMD combinations and SCD (14–16). Currently, it is unknown how much CMM impacts SCD risk.

### 1.2 Goals of investigation

To bridge these research gaps, this study aimed to evaluate the associations between CMM and SCD, using longitudinal EHR-derived data from older adults in the community. By examining the relationships between CMM and SCD, we hope to optimize the prevention of SCD, planning and the delivery of healthcare services for older adults with CMDs.

## 2 Methods

This report followed the Strengthening the Reporting of Observational Studies in Epidemiology (STROBE) reporting guideline.

### 2.1 Study population

Data for this study was derived from the Data Library of Jinnan study. The Jinnan study was designed as a retrospective, population-based cohort study based on EHRs. The anonymized and encrypted EHRs of four towns including physical examinations and cause-of-death surveillance data were provided by the Tianjin Jinnan District Health Commission. These four towns were located in Jinnan District, Tianjin (117.41 N, 38.92 E). There were 290 thousand people, including about 20% of older adults in four towns.

In line with the *Tianjin Code for Basic Public Health Services*, a standardized national physical examination was recorded by township health centers based on the Community Health Service information system, mainly for the older adults. This was done by substrate medical and health institutions. Approximately 68% of people over the age of 65 had a physical examination and were enrolled into the system every year. The data was collected by community medical and health institutions. To obtain accurate outcomes for all older adults, the study linked physical examination data to cause-of-death surveillance data from the China national cause-of-death surveillance system. All deaths in the national cause-of-death surveillance system are reported online through the cause-of-death registration and reporting information system of the Chinese Center For Disease Control and Prevention, which reviews and verifies data reported by provinces and corrects any errors found (17).

The geriatric community dwellers who attended a physical examination from 2017 to 2022 were included in the study. We excluded patients with missing data on outcomes, exposures, or primary covariates. In this study, the first physical examination data of geriatric community dwellers was taken as the baseline data.

### 2.2 Ascertainment of cardiometabolic multimorbidity

In this study, CMM in the baseline data was exposure. CMM was defined as the presence of ≥1 of the following CMDs based on hypertension: coronary heart disease (CHD), stroke, or diabetes (4). Ascertainment of HT, CHD, DM and stroke was by reported physician diagnosis, medication history, via verbal interview.

### 2.3 Ascertainment of outcomes

The primary outcome was SCD. Based on the Framingham study criteria, SCD was defined as death caused by coronary heart disease (definite myocardial infarction, coronary insufficiency, or angina pectoris) within one hour of the onset of symptoms with no other probable cause of death indicated from cause-of-death surveillance data (18). Suspected SCD events were adjudicated by a panel of three trained physicians who applied SCD criteria, using physical examination and cause-of-death surveillance data. Inter-rater reliability values were calculated using Cohen’s Kappa. The Cohen’s kappa was above 0.7. We reviewed SCD events that occurred after January 2017 (baseline) and before December 2022. All-cause mortality was a secondary outcome.

### 2.4 Covariates

Based on existing prior literature and guidelines, the research team’s prior foundational studies, and expert opinions from clinicians and health management professionals, candidate variables were enrolled in this study based on demographics (including gender and age), risk factors (including heart rate, body mass index (BMI), waist, systolic blood pressure (SBP), diastolic blood pressure (DBP), physical activity, current smoking status, serum measures (fasting blood glucose (FBG), triglyceride (TG), total cholesterol (TC), serum creatinine (Scr), blood urea nitrogen (BUN) and total bilirubin (TBIL)), and Electrocardiograph (ECG) (including QTc prolongation and ST wave abnormality) (13, 14). The candidate predictors were listed in the **Supplementary Materials**.

### 2.5 Sensitivity analyses

We performed several sensitivity analyses to test how the results depended on the disease definition, study population, and confounding factors. First, we tested an alternative definition of CMM, which was the presence of ≥1 of the following CMDs: CHD, stroke, or DM. Second, we excluded deaths occurring in the first 2-years of follow-up. Third, we estimated the associations between the different CMDs combinations and SCD, additionally adjusting for ECG at baseline.

### 2.6 Statistical analyses

Continuous variables were expressed as the mean ± standard deviation (SD) or the median (interquartile range). Categorical variables were sorted by frequency (percentages). Baseline characteristics of geriatric community dwellers are presented by baseline CMM status using the χ^2^ test for categorical variables, analysis of variance for parametric continuous variables, and Kruskal-Wallis test for nonparametric continuous variables.

Cox proportional hazard models were used to evaluate the associations of CMM with SCD and all-cause mortality by CMDs combination and count. The results are expressed as hazard ratios (HRs) and 95% confidence intervals (CIs). The proportional hazards assumption was assessed using Schoenfeld residuals, and no significant violations were noted. First, we assessed the associations between single CMDs and SCD and all-cause mortality. Subsequently, we estimated the associations between the different CMDs combinations and SCD, with no CMDs as the reference category. Third, we assessed the associations between CMDs count (categorized as 0, 1, 2, and ≥3) and SCD, using geriatric community dwellers without CMDs as the reference group. CMDs count was entered as a continuous variable to examine its potential additive dose effect on SCD. Based on prior analyses, covariates were selected *a priori* (13), and multivariable modeling was performed with sequential adjustment as follows: model 1 unadjusted; model 2 adjusted for age and gender; model 3: model 2 plus BMI, physical activity, current smoking status, SBP, and DBP; model 4: model 3 plus FBG, TC, TG, BUN, TBIL, and Scr; model 5: model 4 plus QTc prolongation and ST wave abnormality. Finally, the survival curves and subgroup analysis (model 1) were plotted by the number of CMM. Statistical significance was defined as 2-sided α < 0.05 in the main analysis. Analyses were performed using R version 4.3.2.

## 3 Results

A total of 55,184 geriatric community dwellers attended a physical examination from 2017 to 2022. 54 geriatric community dwellers were excluded for missing data on outcomes, exposures, or primary covariates. After these exclusions, 55,130 geriatric community dwellers were included in the analysis (**Supplementary Figure S1**).

**Table 1** shows the characteristics of geriatric community dwellers and prevalence of CMM at baseline. The median (IQR) age was 71 (68, 77) years, and 46.8% of geriatric community dwellers were male. The prevalence of CMM was 25.4%, in which 3.6% had ≥3 CMDs. In geriatric community dwellers with CMM, the combination of HT and DM was the most common (17.0%). Individuals with CMM were older, more likely to be female, and had a higher prevalence of cardiovascular risk factors (eg, higher heart rate, higher BMI, bigger waist size, greater proportion of smokers, higher FBG, TC and TG, lower TBIL and Scr, greater proportion of QTc prolongation and ST wave abnormality) compared to those without CMD. The median follow-up was 5.7 years. During the follow-up, 452 SCDs and 4,657 deaths were documented. Individuals with HT, DM, stroke, or CHD had a higher prevalence of SCD and all-cause mortality (**Supplementary Tables S1, S2**). **Supplementary Tables S3, S4** shows the characteristics of geriatric community dwellers and the prevalence of single CMD at baseline.

**Table 1:** Baseline characteristics of geriatric community dwellers classified by baselinecardiometabolic multimorbidity.

Characteristics	HT + CHD	HT + DM	HT + Stroke	HT + CHD + DM	HT + CHD+ Stroke	HT + DM + Stroke	HT + CHD + DM + Stroke
n (%)	944 (1.7)	9379 (17.0)	1659 (3.0)	677 (1.2)	122 (0.2)	1128 (2.0)	118 (0.2)
Male (%)	369 (39.1)	4115 (43.9)	992 (59.8)	266 (39.3)	65 (53.3)	616 (54.6)	65 (55.1)
Age, years (median (IQR))	77 (71, 82)	72 (68, 77)	74 (70, 80)	75 (70, 80)	77 (73, 81)	74 (70, 79)	76 (70, 82)
Heart rate, bpm (median (IQR))	70 (62, 78)	72 (65, 80)	70 (63, 79)	71 (64, 80)	73 (64, 81)	72 (66, 80)	72 (65, 82)
BMI, kg/m^2^ (median (IQR))	25.6 (23.4, 28.0)	25.8 (23.5, 28.2)	25.9 (23.5, 28.1)	26.1 (23.8, 28.4)	26.3 (24.1, 28.2)	25.8 (23.5, 28.2)	26.0 (24.1, 28.9)
Waist, cm (median (IQR))	90.0 (84.0, 97.0)	90.0 (83.0, 96.0)	89.0 (83.0, 96.0)	90.0 (84.0, 98.0)	92.0 (85.0, 98.0)	89.0 (83.0, 96.0)	91.5 (87.0, 99.8)
SBP (mmHg) (median (IQR))	130.0 (122.0, 138.0)	130.0 (120.0, 138.0)	130.0 (120.0, 138.0)	130.0 (122.0, 138.0)	130.0 (120.0, 138.0)	130.0 (120.0, 138.0)	130.0 (120.0, 138.0)
DBP (mmHg) (median (IQR))	80.0 (74.0, 84.0)	80.0 (74.0, 84.0)	80.0 (76.0, 84.0)	80.0 (74.0, 84.0)	80.0 (76.0, 84.0)	80.0 (74.0, 84.0)	80.0 (72.0, 84.0)
Physical activity (%)	610 (64.6)	6401 (68.2)	1019 (61.4)	416 (61.4)	66 (54.1)	667 (59.1)	58 (49.2)
Smoke (%)	339 (35.9)	3741 (39.9)	894 (53.9)	203 (23.0)	44 (36.1)	514 (45.6)	41 (34.7)
FBG, mmol/L (median (IQR))	5.4 (5.0, 5.9)	7.2 (6.1, 8.7)	5.4 (4.9, 5.8)	7.0 (6.0, 8.4)	5.4 (5.0, 6.0)	7.3 (6.0, 9.1)	7.1 (6.1, 8.8)
TBIL, μmol/L (median (IQR))	12.2 (9.1, 15.9)	12.4 (9.1, 16.6)	12.3 (9.2, 16.7)	11.3 (8.4, 15.3)	12.2 (9.4, 16.7)	11.9 (8.7, 15.9)	12.5 (8.9, 15.7)
Scr, μmol/L (median (IQR))	67.0 (56.5, 79.0)	64.0 (54.0, 77.0)	71.6 (60.6, 85.5)	65.9 (56.0, 80.0)	70.3 (59.0, 85.9)	69.0 (58.0, 84.3)	68.8 (58.0, 83.0)
BUN, mmol/L (median (IQR))	5.4 (4.6, 6.5)	5.4 (4.5, 6.5)	5.6 (4.5, 6.7)	5.6 (4.6, 7.0)	5.5 (4.2, 6.8)	5.6 (4.5, 6.8)	5.5 (4.5, 6.6)
TC, mmol/L (median (IQR))	5.1 (4.3, 5.9)	5.3 (4.6, 6.1)	5.1 (4.4, 5.9)	5.0 (4.3, 6.0)	4.8 (3.9, 5.8)	5.1 (4.2, 5.9)	4.9 (4.1, 5.7)
TG, mmol/L (median (IQR))	1.4 (1.1, 1.9)	1.6 (1.2, 2.3)	1.4 (1.0, 1.9)	1.6 (1.2, 2.2)	1.4 (1.1, 1.8)	1.6 (1.2, 2.3)	1.4 (1.1, 2.0)
QTc prolongation (%)	40 (4.2)	485 (5.2)	76 (4.6)	47 (6.9)	9 (7.4)	70 (6.2)	11 (9.3)
ST wave abnormality	51 (5.4)	302 (3.2)	77 (4.6)	51 (7.5)	9 (7.4)	43 (3.8)	5 (4.2)
SCD (%)	15 (1.6)	160 (1.7)	17 (1.0)	9 (1.3)	2 (1.6)	24 (2.1)	4 (3.4)
All-cause mortality (%)	100 (10.6)	1182 (12.6)	346 (20.9)	102 (15.1)	22 (18.0)	310 (27.5)	28 (23.7)

HT, hypertension; CHD, coronary heart disease; DM, diabetes mellitus; BMI, body mass index; SBP, systolic blood pressure; DBP, diastolic blood pressure; FBG, fasting blood glucose; TBIL, total bilirubin; Scr, serum creatinine; BUN, blood urea nitrogen; TC, total cholesterol; TG, triglyceride.

**Figure 1:**
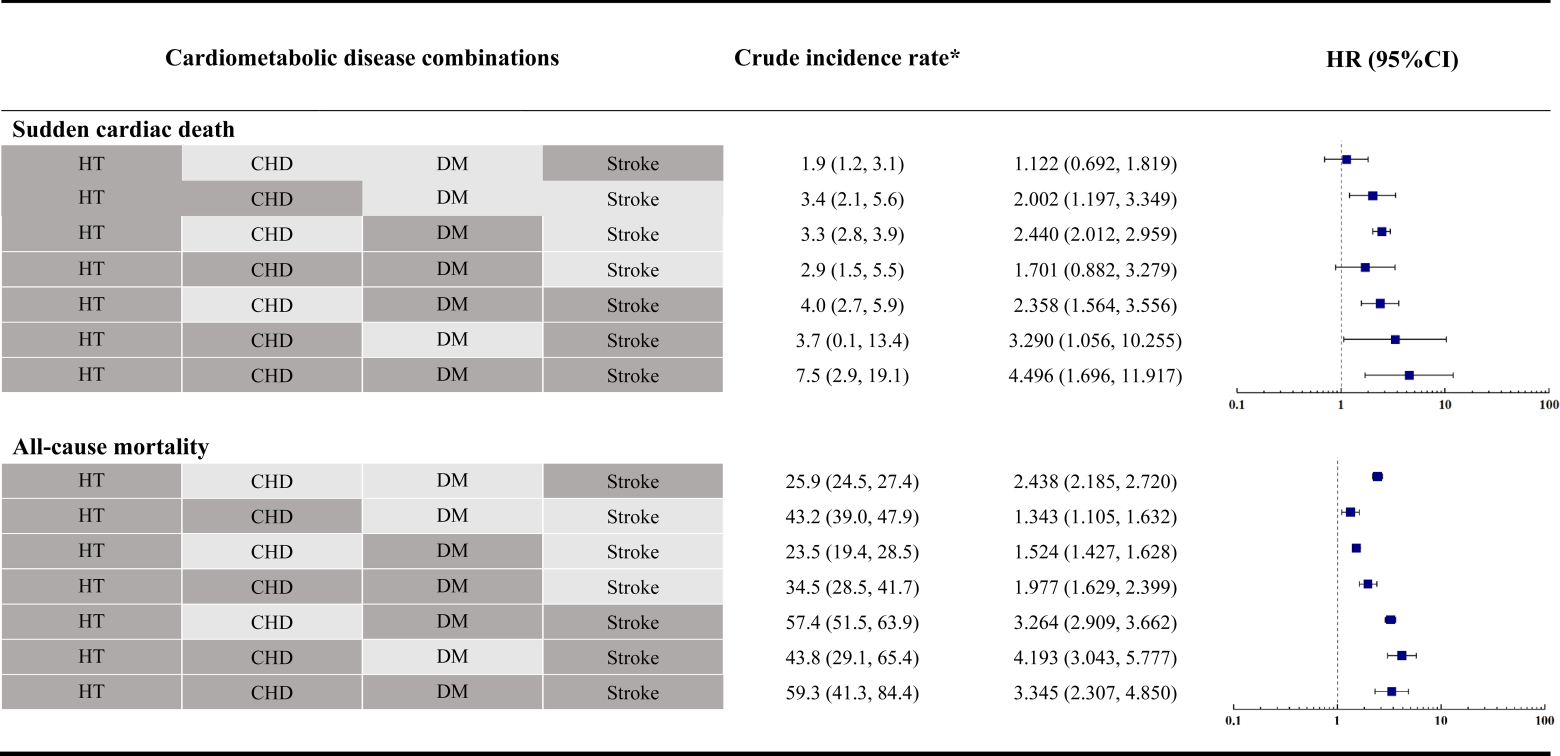
Forest plots of the association of the pattern of cardiometabolic multimorbidity with sudden cardiac death and all-cause mortality. *: No. per 1000 person-years (95% CI).

The crude mortality rates for SCD were 1.9 (1.2, 3.1) per 1000 person-years in overall older adults with HT and stroke, 3.4 (2.1, 5.6) per 1000 person-years in older adults with HT and CHD, 3.3 (2.8, 3.9) per 1000 person-years in older adults with HT and DM. 2.9 (1.5, 5.5) per 1000 person-years in older adults with HT, CHD, and DM, 4.0 (2.7, 5.9) per 1000 person-years in older adults with HT, DM, and stroke, 3.7 (0.1, 13.4) per 1000 person-years in older adults with HT, CHD and stroke, 7.5 (2.9, 19.1) per 1000 person-years in older adults with HT, CHD, DM and stroke, respectively. **Figure 1** shows the crude mortality rates for all-cause mortality.

CMDs combinations were associated with a higher risk of SCD, compared with the reference group (**Figure 1**). Geriatric community dwellers with HT, CHD, DM, and stroke were associated with the greatest risk of SCD (HR, 4.496; 95% CI, 1.696, 11.917), followed by those with HT, CHD, and stroke (HR, 3.290; 95% CI, 1.056, 10.255). Geriatric community dwellers with HT, CHD, stroke, and stroke were associated with the greatest risk of all-cause mortality (HR, 4.193; 95% CI, 3.043, 5.777), followed by those with HT, CHD, and stroke (HR, 3.345; 95% CI, 2.307, 4.850). A statistical model with more conservative covariate adjustment was also conducted and results are presented in **Table 2**.

**Table 2:** The association of the pattern of cardiometabolic multimorbidity with sudden cardiac death and all-cause mortality.

Outcome	Non-CMD	HT+CHD	HT+DM	HT+Stroke	HT+CHD+DM	HT+CHD+Stroke	HT+DM+Stroke	HT+CHD+DM+Stroke
SCD								
Model, HR (95% CI)								
1	Reference	2.002 (1.197, 3.349)	2.440 (2.012, 2.959)	1.122 (0.692, 1.819)	1.701 (0.882, 3.279)	3.290 (1.056, 10.255)	2.358 (1.564, 3.556)	4.496 (1.696, 11.917)
2	Reference	1.419 (0.847, 2.379)	2.542 (2.096, 3.084)	0.862 (0.530, 1.401)	1.431 (0.739, 2.769)	1.650 (0.411, 6.621)	2.031 (1.346, 3.064)	3.434 (1.315, 8.964)
3	Reference	1.454 (0.867, 2.437)	2.572 (2.120, 3.121)	0.811 (0.499, 1.319)	1.416 (0.731, 2.743)	1.635 (0.407, 6.566)	1.901 (1.259, 2.869)	3.098 (1.188, 8.075)
4	Reference	1.574 (0.937, 2.645)	2.163 (1.747, 2.678)	0.890 (0.546, 1.450)	1.088 (0.559, 2.116)	1.824 (0.454, 7.327)	1.363 (0.892, 2.083)	2.200 (0.822, 5.892)
5	Reference	1.544 (0.919, 2.594)	2.178 (1.760, 2.694)	0.880 (0.540, 1.433)	1.014 (0.521, 1.974)	1.771 (0.441, 7.117)	1.311 (0.855, 2.012)	2.218 (0.824, 5.970)
All-cause mortality								
Model, HR (95% CI)								
1	Reference	1.343 (1.105, 1.632)	1.524 (1.427, 1.628)	2.438 (2.185, 2.720)	1.977 (1.629, 2.399)	4.193 (3.043, 5.777)	3.264 (2.909, 3.662)	3.345 (2.307, 4.850)
2	Reference	0.848 (0.695, 1.034)	1.636 (1.531, 1.747)	1.839 (1.648, 2.052)	1.569 (1.289, 1.909)	1.692 (1.113, 2.573)	2.845 (2.535, 3.192)	2.578 (1.797, 3.698)
3	Reference	0.880 (0.721, 1.073)	1.662 (1.555, 1.776)	1.714 (1.536, 1.914)	1.574 (1.293, 1.917)	1.668 (1.097, 2.536)	2.700 (2.406, 3.031)	2.262 (1.560, 3.281)
4	Reference	0.901 (0.739, 1.100)	1.485 (1.379, 1.599)	1.816 (1.626, 2.029)	1.345 (1.103, 1.640)	1.750 (1.151, 2.663)	2.312 (2.052, 2.606)	1.894 (1.305, 2.750)
5	Reference	0.897 (0.735, 1.095)	1.490 (1.384, 1.604)	1.800 (1.611, 2.011)	1.014 (0.521, 1.974)	1.735 (1.141, 2.640)	2.305 (2.045, 2.599)	1.885 (1.298, 2.736)

HT, hypertension; CHD, coronary heart disease; DM, diabetes mellitus; HR, hazard ratio.

Model 1 unadjusted; model 2 adjusted for age and gender; model 3: model 2 plus BMI, physical activity, current smoking status, SBP and DBP; model 4: model 3 plus FBG, T, TG, BUN, TBIL and Scr; model 5: model 4 plus QTc prolongation and ST wave abnormality.

**Figure 2:**
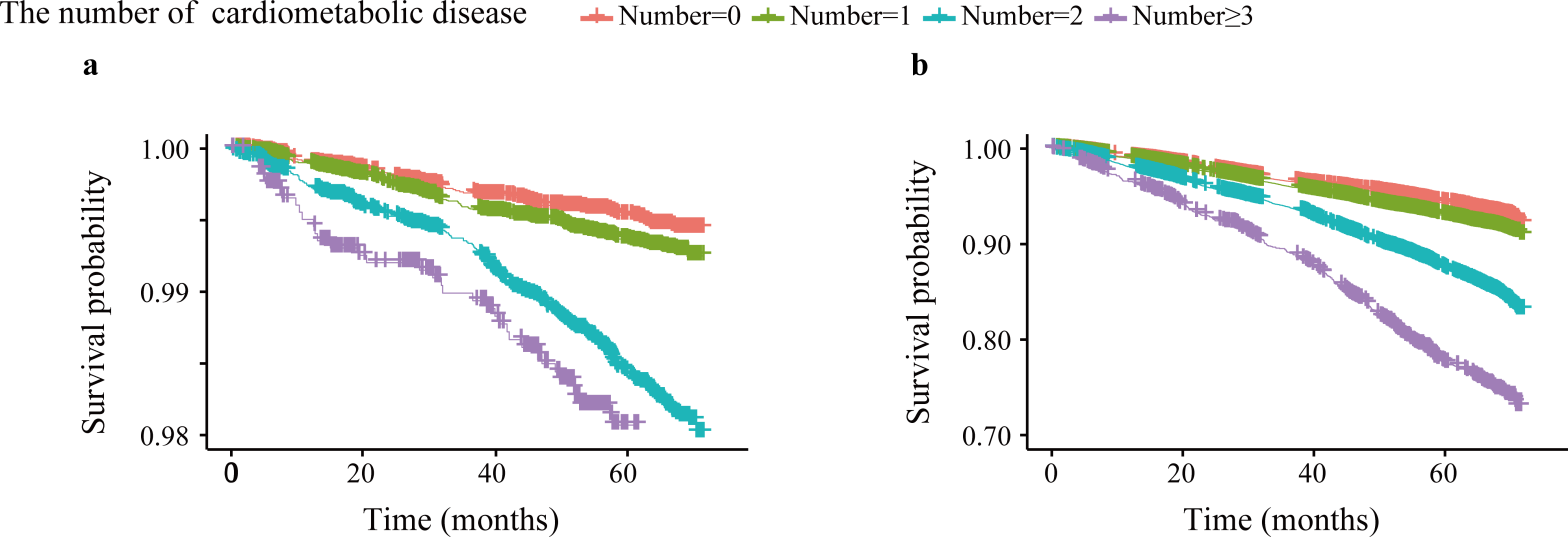
Survival curves for sudden cardiac death and all-cause mortality by the number of cardiometabolic multimorbidity. **(a)**: sudden cardiac death; **(b)**: all-cause mortality.

The results of associations between CMDs and all-cause mortality, and CMDs count are shown in **Figure 2**; **Supplementary Figure S2**. There was an unadjusted additive dose effect of increasing CMDs numbers on SCD (HR, 1.787; 95% CI, 1.606, 1.987). The demographic, risk factors, serum measures and ECG-adjusted HR for SCD was 1.488 (1.327, 1.668) for geriatric community dwellers with an increasing number of CMDs. There was an unadjusted additive dose effect of increasing number of CMDs on all-cause mortality (HR, 1.628; 95% CI, 1.574, 1.683). The demographic, risk factors, serum measures and ECG-adjusted HR for all-cause mortality were 1.473 (1.420, 1.527) for geriatric community dwellers with an increasing number of CMDs. Our findings remained robust after sensitivity analyses (**Supplementary Figures S3, S4**).

In the stratified analysis, older adults with 65≤age<75 (HR, 2.114; 95% CI, 1.747, 2.559) and BMI<24 (HR, 1.921; 95% CI, 1.607, 2.297) showed increased CMM risk. In addition, positive associations between CMM and all-cause mortality were more pronounced in non-smokers (HR, 1.721; 95% CI, 1.640, 1.806) (**Table 3**).

**Table 3:** The association of the number of cardiometabolic multimorbidity with sudden cardiac death and all-cause mortality stratified by potential risk factors.

	Sudden cardiac death	all-cause mortality
Model, HR (95% CI)		
Gender		
Male	1.771 (1.532, 2.047)	1.608 (1.537, 1.682)
Female	1.801 (1.541, 2.106)	1.652 (1.571, 1.737)
Age, years		
65 ≤ Age < 75	2.114 (1.747, 2.559)	1.692 (1.589, 1.803)
75 ≤ Age < 85	1.591 (1.358, 1.864)	1.564 (1.486, 1.645)
Age≥85	1.306 (1.044, 1.633)	1.302 (1.222, 1.389)
BMI		
BMI < 24	1.921 (1.607, 2.297)	1.614 (1.525, 1.710)
BMI ≥ 24	1.730 (1.515, 1.976)	1.645 (1.579, 1.715)
Physical activity		
No	1.866 (1.607, 2.167)	1.747 (1.667, 1.832)
Yes	1.676 (1.443, 1.946)	1.502 (1.434, 1.574)
Smoke		
No	1.958 (1.690, 2.268)	1.721 (1.640, 1.806)
Yes	1.627 (1.394, 1.899)	1.563 (1.492, 1.638)
TC, mmol/L		
<5.20	1.667 (1.440, 1.930)	1.591 (1.520, 1.665)
≥5.20	1.912 (1.638, 2.232)	1.652 (1.572, 1.736)
TG, mmol/L		
<1.7	1.718 (1.506, 1.961)	1.631 (1.568, 1.698)
≥1.7	1.956 (1.627, 2.351)	1.752 (1.646, 1.866)
QTc prolongation		
No	1.773 (1.587, 1.981)	1.629 (1.574, 1.687)
Yes	1.712 (1.153, 2.543)	1.431 (1.249, 1.639)
ST wave abnormality		
No	1.773 (1.586, 1.982)	1.613 (1.559, 1.670)
Yes	1.670 (1.045, 2.202)	1.591 (1.376, 1.840)

HR, hazard ratio; BMI, body mass index; TC, total cholesterol; TG, triglyceride.

## 4 Discussion

We found the prevalence of CMM was approximately 25% in geriatric community dwellers in our population. HT and DM was the most prevalent combination of CMM. For single CMDs, DM was associated with the greatest risk of SCD. For CMM, the risk of SCD varied by the pattern of CMM, and were significantly higher with increasing numbers of cardiometabolic conditions (DM, stroke, and CHD) among geriatric community dwellers, even after adjusting for established cardiovascular risk factors. Given the growing challenges of multimorbidity among older adults living in the community, our study holds important implications for public health.

Although, CMM studies have been conducted in Western countries, health concern remains poorly explored in China. With a dense population and an aging society, CMM is notably prevalent among its older population, substantially contributing to the burden on the Chinese public health system. Our study revealed that one in five older adults had CMM. A systematic review of nine published studies in China reported the prevalence of multimorbidity among those aged ≥ 60 years ranging from 6.4% (95% CI: 5.1 to 8.0) to 76.5% (95% CI: 73.6 to 79.2) (19). However, most of the included studies considered morbidities in addition to CMDs and only reported prevalence based on the number of diseases, which prevented us from making direct comparisons. Previous studies have examined that the overall crude prevalence of CMM was 11.5% in Chinese adults aged 35-75 based on a population-based screening project in Southern China (20), and 6.3% of Chinese adults aged 35-75 in China Kadoorie Biobank had CMM (21). The findings may not be generalizable to the wider population. Our study provided additional evidence for CMM studies in Chinese older adults and the urgency for CMD prevention, considering population ageing (22).

Our results indicate that the associations between cardiovascular disease and DM, and mortality are multiplicative. HT is the principal cause of stroke, a major risk factor for DM, CHD, and SCD. It is estimated that HT prevalence was about 40%, which was broadly consistent with estimates from previous studies (23). Previous studies have indicated that older adults with multimorbidity are most likely to have HT and at least one other chronic illness (4). Further, one study found that the risk of all-cause mortality increased significantly (from 7% to 30%) after the progression of CMM in patients with HT (9). SCD resulting from ventricular tachycardia and fibrillation (VT/VF) is closely linked to HT. Left ventricular hypertrophy, a common consequence of HT, increases the risk of ventricular arrhythmias, which in turn elevates the likelihood of SCD.

The estimated prevalence of DM in our study was 4.4%, which was broadly consistent with estimates from national surveillance (24). In addition, we found DM, as a kind of CMD, was associated with the greatest risk of SCD (25). A number of epidemiologic studies have shown that the risk of SCD is higher among patients with DM compared with those without this condition (26). In a meta-analysis of individuals over 50 years of age, people with DM have a higher risk of SCD than people without DM (risk ratio, 2.02; 95% CI, 1.81-2.25) (27). Consequently, our findings highlight the importance of preventing cardiovascular disease in people with DM, as well as preventing DM in people with cardiovascular disease. DM contributes to SCD through a combination of structural, electrical, and inflammatory changes in the heart, as well as through its effects on coronary and autonomic function.

With the higher prevalence of risk factors, the incidence of CAD and associated mortality is increasing in China. The results of published literature indicate that CAD accounts for approximately 80% of all SCD cases, and that the incidence of CAD increases with increasing age (28). This demonstrates the importance of cardiovascular disease risk factor control to prevent SCD.

According to recent publications, risk for all-cause mortality was the highest for people with stroke (HR, 1.74; 95% CI, 1.24-2.42) for people with only 1 CMD (29). In our study, stroke was more associated with all-cause mortality than SCD. It is unclear why stroke mortality is higher, but it may be attributed to a higher stroke incidence and mortality after strokes (30).

The estimated prevalence of CMM in our study was broadly consistent with estimates from previous studies. For example, about 1.3% of participants aged ≥ 60 years had multimorbidity for DM and CHD, and the prevalence of HT, DM and stroke was 2.0% in our study (31). Moreover, our study indicated a positive association between CMDs, and SCD and all-cause mortality risks. The Emerging Risk Factors Collaboration (ERFC) pooled 91 prospective cohorts conducted in North America, Europe, and Australia. Compared with no CMDs, the HR (95% CIs) for concurrent three CMDs at baseline was 6.0 (5.0, 7.1) (2). Compared with those without CMDs at baseline, the HR (95% CIs) for concurrent three CMDs was 3.22 (3.15, 3.30) in the Clinical Practice Research Datalink (CPRD) study (32). CHD, DM and stroke were associated with the highest mortality rates among Black adults in the Jackson Heart Study (JHS) (HR, 3.68; 95% CI, 1.96-6.93) (29). Based on the China Kadoorie Biobank study, participants with three CMDs at baseline had an adjusted HR between CMM and all-cause mortality and circulatory system diseases of 2.93 (2.80, 3.07) and 5.05 (4.74, 5.37) respectively (21). The HRs might be explained by differences in race, healthcare services, and economic levels. This suggests that it is important to consider differences when developing strategies to improve public health. For health professionals, a holistic assessment of this information has the potential to improve disease management. The multiplicative increased risk of SCD is a call to action to prevent the development of cardiometabolic disease and advance the treatment of care of those with known cardiometabolic morbidities. Additionally, a lifestyle intervention program and effective treatment for prevalent CMDs should be implemented to prevent the occurrence of CMM and reduce the risk of SCD and mortality among patients with CMDs. Future studies should explain why certain cardiometabolic diseases (CMDs) combinations may have synergistic effects leading to higher SCD risk.

The strength of this study was that it was a large-scale, comprehensive study under real-world circumstances using EHR data. To the best of our knowledge, this is the first comprehensive population-based cohort investigating the association of CMM and SCD among general older adults. Moreover, sensitivity analysis was conducted in our study to confirm the robustness of our results. We were able to comprehensively analyze the associations between single CMDs and different CMM patterns, and SCD risk.

Despite these strengths, our study remains limited due to several shortcomings. First, self-reported disease diagnoses could underestimate the disease prevalence. Second, multimorbidity might be affected by detection biases (ie, one disease is detected and then others are tested because of it). Third, CMDs commonly have an insidious onset. Therefore, the registration date recorded in our study may be later than actual onset, and the accurate time of disease onset was not available. We could only approximate this information using the first registration time for these diseases. The short follow-up period could potentially bias the results, particularly for diseases with a longer progression period. Information on medication use and risk control was also not available. Fourth, this study had a relatively short follow-up period and the long-term effect of CMM needs to be further evaluated. Fifth, unknown or unmeasured factors may induce residual confounding, and these factors may affect our results (such as medication use, lifestyle interventions, healthcare systems, socioeconomic factors and genetic predispositions). Sixthly, The study design is observational, so causality cannot be established. Finally, it should be acknowledged that the study’s findings are based on research conducted solely on older Chinese adults and, therefore, may not be generalizable to other cultural or ethnic populations.

## 5 Conclusion

In this cohort study, an increasing number of CMM was associated with a multiplicative increase in risk of SCD and all-cause mortality among geriatric community dwellers, with a greater magnitude of association for SCD.

## Data Availability

The original contributions presented in the study are included in the article/**Supplementary Material**. Further inquiries can be directed to the corresponding authors.
