# Supplementary material for: Cardiometabolic multimorbidity and the risk of sudden cardiac death among geriatric community dwellers using longitudinal EHR-derived data
Source: Front Endocrinol (Lausanne). 2025 Apr 25;16:1515495. doi: 10.3389/fendo.2025.1515495 (PMC12061703; doi:10.3389/fendo.2025.1515495)
Supplement: Supplementary file 1 [file DataSheet1.doc]

SUPPLEMENTAL MATERIALS

# Covariates

Gender, age, physical activity and current smoking status were determined by self-report. Height, weight and waist were measured by standard protocol; and body mass index (BMI) was calculated as weight (in kilograms) divided by height (in meters) squared. Blood pressure (systolic and diastolic) and heart rate were measured in seated participants after a 5-minute rest. Serum measurements were made from fasting serum and plasma. QTc prolongation and ST wave abnormality were determined from a 12-lead electrocardiogram. QT interval was corrected for heart rate according to the Bazett formula, and QTc interval >450ms for men and >460ms for women was considered prolonged. An ST-wave abnormality was defined as having Minnesota codes 4-1 through 4-3 or codes 4-2 through 5-3.

# Captions and legends of supplemental table and figure

**Table S1 Baseline Characteristics of older adults by sudden cardiac death**

BMI: body mass index, SBP: systolic blood pressure, DBP: diastolic blood pressure, FBG: fasting blood glucose, TBIL: total bilirubin, Scr: serum creatinine, BUN: blood urea nitrogen, TC: total cholesterol, TG: triglyceride.

**Table S2 Baseline Characteristics of older adults by all-cause mortality**

BMI: body mass index, SBP: systolic blood pressure, DBP: diastolic blood pressure, FBG: fasting blood glucose, TBIL: total bilirubin, Scr: serum creatinine, BUN: blood urea nitrogen, TC: total cholesterol, TG: triglyceride.

**Table S3 Baseline characteristics of participants classified by baseline cardiometabolic disease**

HT: hypertension, CHD: coronary heart disease, DM: diabetes mellitus, BMI: body mass index, SBP: systolic blood pressure, DBP: diastolic blood pressure, FBG: fasting blood glucose, SGPT: serum glutamic pyruvic transaminase, SGOT: serum glutamic-oxaloacetic transaminase, TBIL: total bilirubin, Scr: serum creatinine, BUN: blood urea nitrogen, TC: total cholesterol, TG: triglyceride.

**Table S4 Association of cardiometabolic disease with sudden cardiac death and all-cause mortality**

*: No. per 1000 person-years (95% CI). HT: hypertension, CHD: coronary heart disease, DM: diabetes mellitus, HR: hazard ratio.

Model 1 unadjusted; model 2 adjusted for age and gender; model 3: model 2 plus body mass index, physical activity, current smoking status, systolic blood pressure and diastolic blood pressure; model 4: model 3 plus fasting blood glucose, total cholesterol, triglyceride, blood urea nitrogen, total bilirubin and serum creatinine; model 5: model 4 plus QTc prolongation and ST wave abnormality.

**Figure S1 Flow chart for participant selection**

**Figure S2 Forest plots of the association of the number of cardiometabolic multimorbidity with sudden cardiac death and all-cause mortality**

**Figure S3 Forest plots of the association of the number of cardiometabolic multimorbidity with sudden cardiac death and all-cause mortality in sensitivity analyses (another definition of CMM)**

**Figure S4 Forest plots of the association of the number of cardiometabolic multimorbidity with sudden cardiac death and all-cause mortality in sensitivity analyses (excluding the deaths occurring in the first 2-year of follow-up)**

Table S1 Baseline Characteristics of older adults by sudden cardiac death

|  | **Overall** | **Non-SCD** | **SCD** | ***p*** |
| --- | --- | --- | --- | --- |
| n | 55130 | 54678 | 452 |  |
| Gender |  |  |  |  |
| Male | 25782 (46.8) | 25545 (46.7) | 237 (52.4) | 0.017 |
| Female | 29348 (53.2) | 29133 (53.3) | 215 (47.6) |  |
| Age, years | 71.0 (68.0, 77.0) | 71.0 (68.0, 77.0) | 78.0 (73.0, 83.0) | <0.001 |
| Heart rate, bpm | 70.0 (63.0, 78.0) | 70.0 (63.0, 78.0) | 72.0 (65.0, 81.0) | <0.001 |
| BMI, kg/m^2^ | 25.4 (23.2, 27.8) | 25.4 (23.2, 27.8) | 25.3 (23.3, 27.6) | 0.614 |
| Waist, cm | 89.0 (83.0, 95.0) | 89.0 (83.0, 95.0) | 89.0 (83.0, 96.0) | 0.884 |
| SBP (mmHg) | 130.0 (120.0, 138.0) | 130.0 (120.0, 138.0) | 130.0 (120.0, 138.0) | 0.643 |
| DBP (mmHg) | 80.0 (74.0, 84.0) | 80.0 (74.0, 84.0) | 80.0 (76.0, 84.0) | 0.868 |
| Physical activity (%) |  |  |  |  |
| No | 18099 (32.8) | 17886 (32.7) | 213 (47.1) | <0.001 |
| Yes | 37031 (67.2) | 36792 (67.3) | 239 (52.9) |  |
| Smoke (%) |  |  |  |  |
| No | 32461 (58.9) | 32226 (58.9) | 235 (52.0) | 0.003 |
| Yes | 22669 (41.1) | 22452 (41.1) | 217 (48.0) |  |
| Stroke (%) |  |  |  |  |
| No | 51768 (93.9) | 51368 (93.9) | 400 (88.5) | <0.001 |
| Yes | 3362 (6.1) | 3310 (6.1) | 52 (11.5) |  |
| Diabetes (%) |  |  |  |  |
| No | 41237 (74.8) | 41017 (75.0) | 220 (48.7) | <0.001 |
| Yes | 13893 (25.2) | 13661 (25.0) | 232 (51.3) |  |
| Hypertension (%) |  |  |  |  |
| No | 21395 (38.8) | 21284 (38.9) | 111 (24.6) | <0.001 |
| Yes | 33735 (61.2) | 33394 (61.1) | 341 (75.4) |  |
| FBG, mmol/L | 5.6 (5.1, 6.3) | 5.6 (5.1, 6.3) | 5.7 (5.0, 7.3) | 0.022 |
| TBIL, μmol/L | 12.8 (9.4, 16.9) | 12.8 (9.4, 16.9) | 10.8 (8.2, 14.7) | <0.001 |
| Scr, μmol/L | 65.6 (55.5, 76.9) | 65.5 (55.4, 76.7) | 69.0 (58.0, 85.0) | <0.001 |
| BUN, mmol/L | 5.3 (4.4, 6.3) | 5.3 (4.4, 6.3) | 6.0 (5.0, 7.4) | <0.001 |
| TC, mmol/L | 5.3 (4.6, 6.0) | 5.3 (4.6, 6.0) | 5.2 (4.3, 6.0) | 0.138 |
| TG, mmol/L | 1.4 (1.0, 2.0) | 1.4 (1.0, 2.0) | 1.4 (1.0, 2.1) | 0.304 |
| QTc prolongation |  |  |  |  |
| No | 52935 (96.0) | 52516 (96.0) | 419 (92.7) | <0.001 |
| Yes | 2195 (4.0) | 2162 (4.0) | 33 (7.3) |  |
| ST wave abnormality |  |  |  |  |
| No | 53684 (97.4) | 53271 (97.4) | 413 (91.4) | <0.001 |
| Yes | 1446 (2.6) | 1407 (2.6) | 39 (8.6) |  |

BMI: body mass index, SBP: systolic blood pressure, DBP: diastolic blood pressure, FBG: fasting blood glucose, TBIL: total bilirubin, Scr: serum creatinine, BUN: blood urea nitrogen, TC: total cholesterol, TG: triglyceride.

Table S2 Baseline Characteristics of older adults by all-cause mortality

|  | **Overall** | **Alive** | **Deceased all-cause mortality** | ***p*** |
| --- | --- | --- | --- | --- |
| n | 55130 | 50473 | 4657 |  |
| Gender (%) |  |  |  |  |
| Male | 25782 (46.8) | 23257 (46.1) | 2525 (54.2) | <0.001 |
| Female | 29348 (53.2) | 27216 (53.9) | 2132 (45.8) | |
| Age, years (median (IQR)) | 71.0 (68.0, 77.0) | 71.0 (67.0, 76.0) | 79.0 (74.0, 85.0) | <0.001 |
| Heart rate, bpm (median (IQR)) | 70.0 (63.0, 78.0) | 69.0 (63.0, 77.0) | 73.0 (65.0, 81.0) | <0.001 |
| BMI, kg/m^2^ (median (IQR)) | 25.4 (23.2, 27.8) | 25.4 (23.2, 27.8) | 25.5 (23.2, 27.9) | 0.315 |
| Waist, cm (median (IQR)) | 89.0 (83.0, 95.0) | 89.0 (83.0, 95.0) | 89.0 (82.0, 96.0) | 0.925 |
| SBP (mmHg) (median (IQR)) | 130.0 (120.0, 138.0) | 130.0 (120.0, 138.0) | 130.0 (120.0, 138.0) | 0.007 |
| DBP (mmHg) (median (IQR)) | 80.0 (74.0, 84.0) | 80.0 (74.0, 84.0) | 80.0 (74.0, 84.0) | 0.009 |
| Physical activity (%) |  |  |  |  |
| No | 18099 (32.8) | 15924 (31.5) | 2175 (46.7) | <0.001 |
| Yes | 37031 (67.2) | 34549 (68.5) | 2482 (53.3) | |
| Smoke (%) |  |  |  |  |
| No | 32461 (58.9) | 30225 (59.9) | 2236 (48.0) | <0.001 |
| Yes | 22669 (41.1) | 20248 (40.1) | 2421 (52.0) | |
| Stroke (%) |  |  |  |  |
| No | 51768 (93.9) | 47879 (94.9) | 3889 (83.5) | <0.001 |
| Yes | 3362 (6.1) | 2594 (5.1) | 768 (16.5) |  |
| Diabetes (%) |  |  |  |  |
| No | 41237 (74.8) | 38528 (76.3) | 2709 (58.2) | <0.001 |
| Yes | 13893 (25.2) | 11945 (23.7) | 1948 (41.8) | |
| Hypertension (%) |  |  |  |  |
| No | 21395 (38.8) | 20100 (39.8) | 1295 (27.8) | <0.001 |
| Yes | 33735 (61.2) | 30373 (60.2) | 3362 (72.2) | |
| FBG, mmol/L (median (IQR)) | 5.6 (5.1, 6.3) | 5.6 (5.1, 6.3) | 5.6 (5.0, 6.7) | 0.68 |
| TBIL, μmol/L (median (IQR)) | 12.8 (9.4, 16.9) | 12.9 (9.5, 16.9) | 11.7 (8.6, 16.2) | <0.001 |
| Scr, μmol/L (median (IQR)) | 65.6 (55.5, 76.9) | 65.0 (55.0, 76.0) | 70.0 (58.0, 85.2) | <0.001 |
| BUN, mmol/L (median (IQR)) | 5.3 (4.4, 6.3) | 5.3 (4.4, 6.2) | 5.8 (4.7, 7.2) | <0.001 |
| TC, mmol/L (median (IQR)) | 5.3 (4.6, 6.0) | 5.3 (4.6, 6.0) | 5.2 (4.4, 6.0) | <0.001 |
| TG, mmol/L (median (IQR)) | 1.4 (1.0, 2.0) | 1.4 (1.0, 2.0) | 1.3 (0.9, 1.9) | <0.001 |
| QTc prolongation (%) |  |  |  |  |
| No | 52935 (96.0) | 48563 (96.2) | 4372 (93.9) | <0.001 |
| Yes | 2195 (4.0) | 1910 (3.8) | 285 (6.1) |  |
| ST wave abnormality |  |  |  |  |
| No | 53684 (97.4) | 49293 (97.7) | 4391 (94.3) | <0.001 |
| Yes | 1446 (2.6) | 1180 (2.3) | 266 (5.7) |  |

BMI: body mass index, SBP: systolic blood pressure, DBP: diastolic blood pressure, FBG: fasting blood glucose, TBIL: total bilirubin, Scr: serum creatinine, BUN: blood urea nitrogen, TC: total cholesterol, TG: triglyceride.

Table S3 Baseline characteristics of participants classified by baseline cardiometabolic disease

| **Characteristic** | **None** | **HT** | **CHD** | **DM** | **Stroke** |
| --- | --- | --- | --- | --- | --- |
| n | 18294 | 19708 | 261 | 2442 | 233 |
| Gender (%) |  |  |  |  |  |
| Male | 8837 (48.3) | 8920 (45.3) | 106 (40.6) | 1190 (48.7) | 138 (59.2) |
| Female | 9457 (51.7) | 10788 (54.7) | 155 (59.4) | 1252 (51.3) | 95 (40.8) |
| Age, years (median (IQR)) | 70 (67, 75) | 72 (68, 77) | 76 (70, 80) | 70 (67, 75) | 74 (71, 80) |
| Heart rate, bpm (median (IQR)) | 68 (63, 76) | 70 (63, 78) | 68 (62, 77) | 71 (64, 79) | 70 (65, 79) |
| BMI, kg/m^2^ (median (IQR)) | 24.9 (22.8, 27.3) | 25.6 (23.4, 28.0) | 25.4 (23.2, 27.6) | 25.4 (23.2, 27.7) | 24.6 (22.9, 27.2) |
| Waist, cm (median (IQR)) | 88.0 (82.0, 94.0) | 89.0 (83.0, 96.0) | 89.0 (84.0, 95.0) | 89.0 (83.0, 96.0) | 87.0 (81.0, 94.0) |
| SBP (mmHg) (median (IQR)) | 130.0 (120.0, 136.0) | 130.0 (120.0, 138.0) | 130.0 (120.0, 136.0) | 130.0 (120.0, 136.0) | 130.0 (120.0, 136.0) |
| DBP (mmHg) (median (IQR)) | 80.0 (74.0, 84.0) | 80.0 (76.0, 84.0) | 80.0 (74.0, 82.0) | 80.0 (74.0, 84.0) | 80.0 (74.0, 84.0) |
| Physical activity (%) |  |  |  |  |  |
| No | 6243 (34.1) | 6102 (31.0) | 85 (32.6) | 738 (30.2) | 83 (35.6) |
| Yes | 12051 (65.9) | 13606 (69.0) | 176 (67.4) | 1704 (69.8) | 150 (64.4) |
| Smoke (%) |  |  |  |  |  |
| No | 11055 (60.4) | 11359 (57.6) | 155 (59.3) | 1449 (59.3) | 106 (45.5) |
| Yes | 7239 (39.6) | 8349 (42.4) | 106 (40.62) | 993 (40.7) | 127 (54.5) |
| FBG, mmol/L (median (IQR)) | 5.4 (5.0, 5.7) | 5.4 (5.0, 5.9) | 5.4 (4.9, 5.9) | 7.3 (6.2, 9.0) | 5.3 (4.9, 5.8) |
| TBIL, μmol/L (median (IQR)) | 13.5 (9.8, 17.0) | 12.7 (9.5, 16.9) | 12.1 (9.1, 16.4) | 12.7 (9.2, 17.3) | 12.8 (9.4, 17.5) |
| Scr, μmol/L (median (IQR)) | 65.7 (56.0, 74.0) | 66.0 (56.0, 78.0) | 64.0 (56.0, 75.0) | 63.0 (53.0, 73.8) | 67.0 (57.0, 77.0) |
| BUN, mmol/L (median (IQR)) | 5.3 (4.4, 6.0) | 5.3 (4.5, 6.4) | 5.2 (4.4, 6.1) | 5.3 (4.4, 6.2) | 5.1 (4.3, 6.5) |
| TC, mmol/L (median (IQR)) | 5.3 (4.7, 5.9) | 5.3 (4.7, 6.0) | 5.0 (4.3, 5.8) | 5.2 (4.6, 6.0) | 5.2 (4.5, 5.8) |
| TG, mmol/L (median (IQR)) | 1.2 (0.9, 1.7) | 1.5 (1.1, 2.0) | 1.3 (1.0, 1.7) | 1.5 (1.0, 2.0) | 1.2 (0.9, 1.7) |
| QTc prolongation (%) |  |  |  |  |  |
| No | 17731 (96.9) | 18950 (96.2) | 234 (89.7) | 2349 (96.2) | 224 (96.1) |
| Yes | 563 (3.1) | 758 (3.8) | 27 (10.3) | 93 (3.8) | 9 (3.9) |
| ST wave abnormality |  |  |  |  |  |
| No | 18005 (98.4) | 19157 (97.2) | 251 (96.2) | 2399 (98.2) | 227 (97.4) |
| Yes | 289 (1.6) | 551 (2.8) | 10 (3.8) | 43 (1.8) | 6 (2.6) |
| SCD (%) |  |  |  |  |  |
| No | 18221 (99.6) | 19598 (99.4) | 260 (99.6) | 2411 (98.7) | 232 (99.6) |
| Yes | 73 (0.4) | 110 (0.6) | 1 (0.4) | 31 (1.3) | 1 (0.4) |
| All-cause mortality (%) |  |  |  |  |  |
| No | 17397 (95.1) | 18436 (93.5) | 234 (89.7) | 2143 (87.8) | 193 (82.8) |
| Yes | 897 (4.9) | 1272 (6.5) | 27 (10.3) | 299 (12.2) | 40 (17.2) |

CHD+stroke, stroke+diabetes and CHD+stroke+diabetes are not included in this part of the analysis. HT: hypertension, CHD: coronary heart disease, DM: diabetes mellitus, BMI: body mass index, SBP: systolic blood pressure, DBP: diastolic blood pressure, FBG: fasting blood glucose, SGPT: serum glutamic pyruvic transaminase, SGOT: serum glutamic-oxaloacetic transaminase, TBIL: total bilirubin, Scr: serum creatinine, BUN: blood urea nitrogen, TC: total cholesterol, TG: triglyceride.

**TABLE S4 Association of cardiometabolic disease with sudden cardiac death and all-cause mortality**

| **Outcome** | **Overall** | **Non-CMD** | **HT** | **CHD** | **DM** | **Stroke** |
| --- | --- | --- | --- | --- | --- | --- |
| **SCD** |  |  |  |  |  |  |
| Crude incidence rate (95% CI)* | 1.7 (1.5, 1.9) | 0.9 (0.7, 1.2) | 2.0 (1.8, 2.2) | 3.2 (2.3, 4.5) | 3.3 (2.9, 3.8) | 3.0 (2.3, 3.9) |
| Model, HR (95% CI) |  |  |  |  |  |  |
| 1 | / | Reference | 1.619 (1.306, 2.006) | 1.967 (1.380, 2.804) | 2.906 (2.417, 3.495) | 1.793 (1.343, 2.394) |
| 2 | / | Reference | 1.467 (1.183, 1.818) | 1.496 (1.048, 2.135) | 2.946 (2.449, 3.543) | 1.422 (1.063, 1.901) |
| 3 | / | Reference | 1.487 (1.199, 1.844) | 1.505 (1.053, 2.151) | 2.949 (2.451, 3.548) | 1.334 (0.997, 1.786) |
| 4 | / | Reference | 1.358 (1.093, 1.687) | 1.417 (0.991, 2.026) | 2.661 (2.152, 3.292) | 1.207 (0.900, 1.618) |
| 5 | / | Reference | 1.321 (1.063, 1.642) | 1.362 (0.952, 1.949) | 2.670 (2.159, 3.301) | 1.176 (0.876, 1.578) |
| **All-cause mortality** |  |  |  |  |  |  |
| Crude incidence rate (95% CI)* | 18.4 (17.9, 18.9) | 9.8 (8.9, 10.8) | 20.5 (19.8, 21.2) | 30.6 (27.3, 34.3) | 29.5 (28.2, 30.8) | 48.3 (45.1, 51.7) |
| Model, HR (95% CI) |  |  |  |  |  |  |
| 1 | / | Reference | 1.368 (1.283, 1.459) | 1.751 (1.557, 1.970) | 2.024 (1.909, 2.145) | 2.933 (2.715, 3.169) |
| 2 | / | Reference | 1.228 (1.152, 1.310) | 1.227 (1.090, 1.381) | 2.116 (1.996, 2.243) | 2.285 (2.114, 2.469) |
| 3 | / | Reference | 1.254 (1.176, 1.337) | 1.248 (1.108, 1.405) | 2.136 (2.015, 2.265) | 2.146 (1.985, 2.320) |
| 4 | / | Reference | 1.195 (1.119, 1.275) | 1.199 (1.064, 1.351) | 2.158 (2.014, 2.312) | 2.056 (1.901, 2.224) |
| 5 | / | Reference | 1.181 (1.106, 1.260) | 1.185 (1.052, 1.335) | 2.165 (2.021, 2.319) | 2.038 (1.884, 2.204) |

*: No. per 1000 person-years (95% CI). HT: hypertension, CHD: coronary heart disease, DM: diabetes mellitus, HR: hazard ratio.

Model 1 unadjusted; model 2 adjusted for age and gender; model 3: model 2 plus body mass index, physical activity, current smoking status, systolic blood pressure and diastolic blood pressure; model 4: model 3 plus fasting blood glucose, total cholesterol, triglyceride, blood urea nitrogen, total bilirubin and serum creatinine; model 5: model 4 plus QTc prolongation and ST wave abnormality.


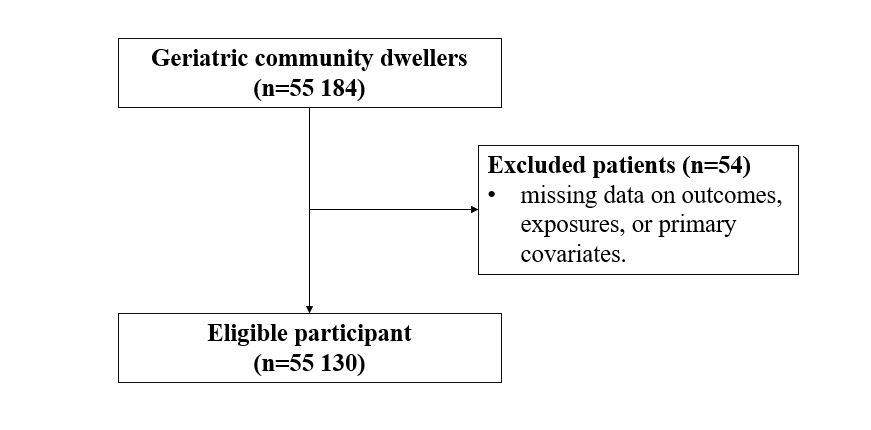


**Figure S1** **Flow chart for participant selection**


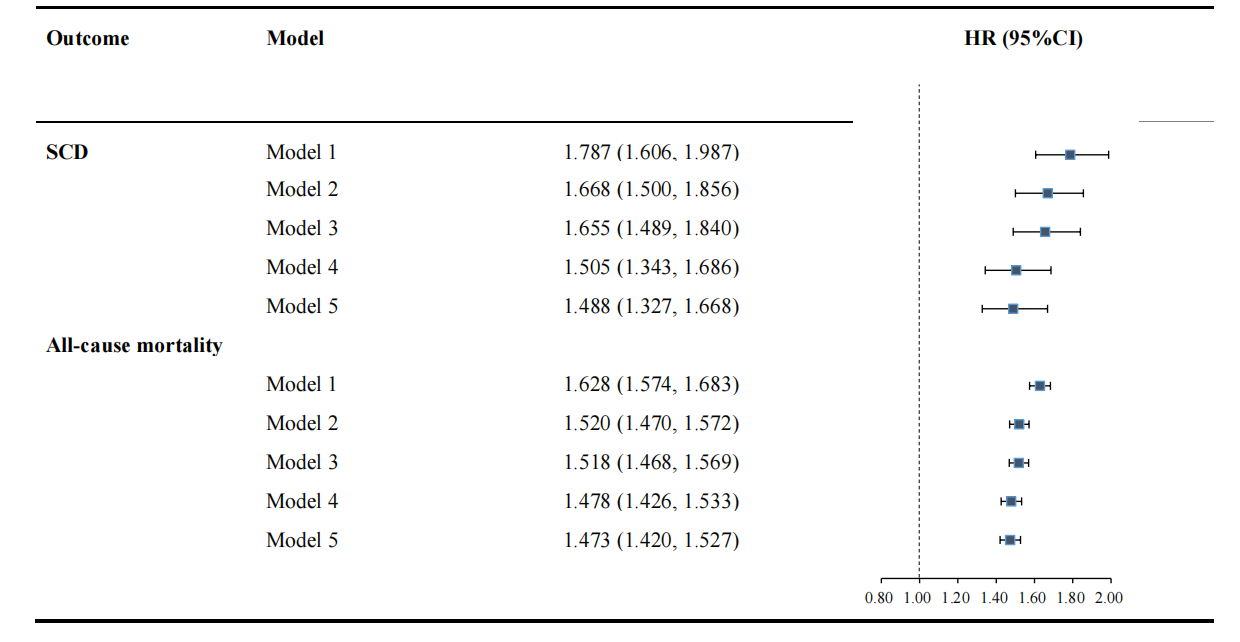


HR: hazard ratio. Model 1 unadjusted; model 2 adjusted for age and gender; model 3: model 2 plus BMI, physical activity, current smoking status, SBP and DBP; model 4: model 3 plus FBG, T, TG, BUN, TBIL and Scr; model 5: model 4 plus QTc prolongation and ST wave abnormality. Reference was the non-CMD group.

**Figure S2 Forest plots of the association of the number of cardiometabolic multimorbidity with s****udden cardiac death and all-cause mortality**


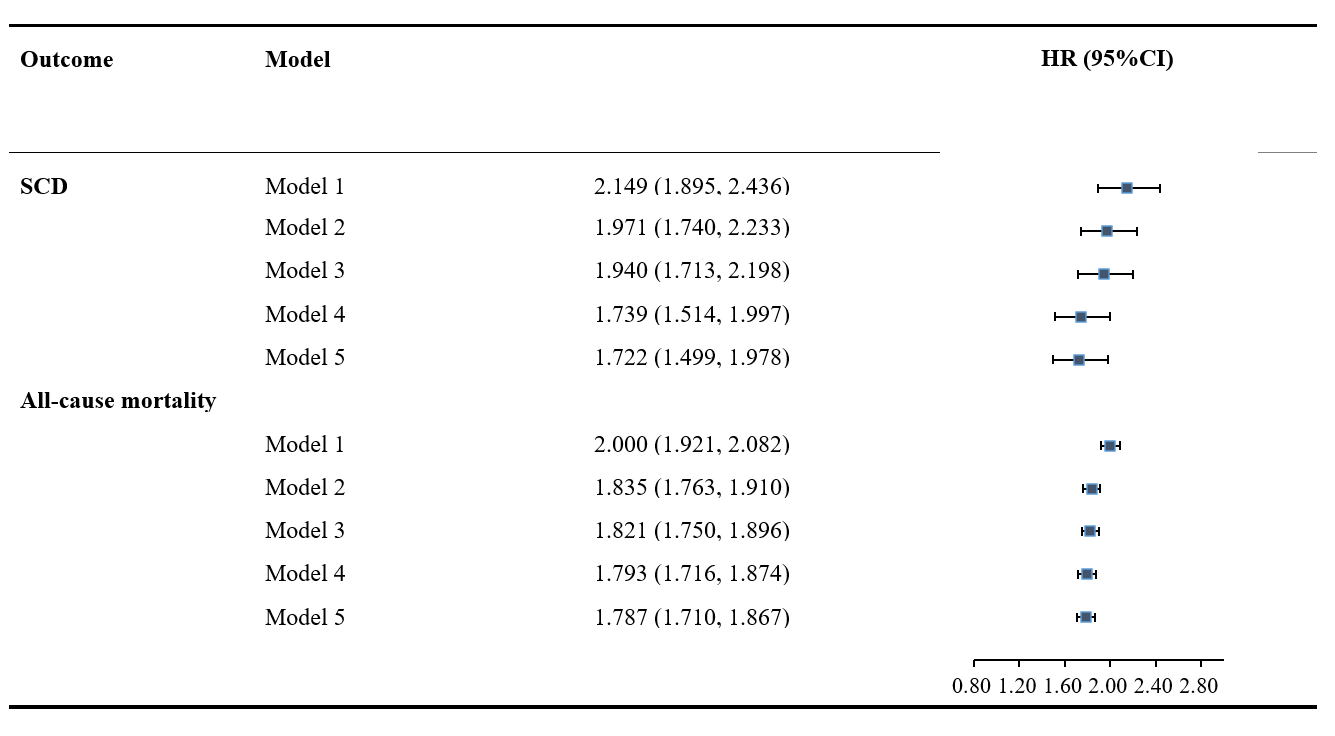


HR: hazard ratio. Model 1 unadjusted; model 2 adjusted for age and gender; model 3: model 2 plus BMI, physical activity, current smoking status, SBP and DBP; model 4: model 3 plus FBG, T, TG, BUN, TBIL and Scr; model 5: model 4 plus QTc prolongation and ST wave abnormality. Reference was the non-CMD group.

The definition of CMM was the presence of ≥1 of the following CMDs: CHD, stroke, or DM

**Figure S3 Forest plots of the association of the number of cardiometabolic multimorbidity with sudden cardiac death and all-cause mortality in sensitivity analyses (another definition of CMM)**


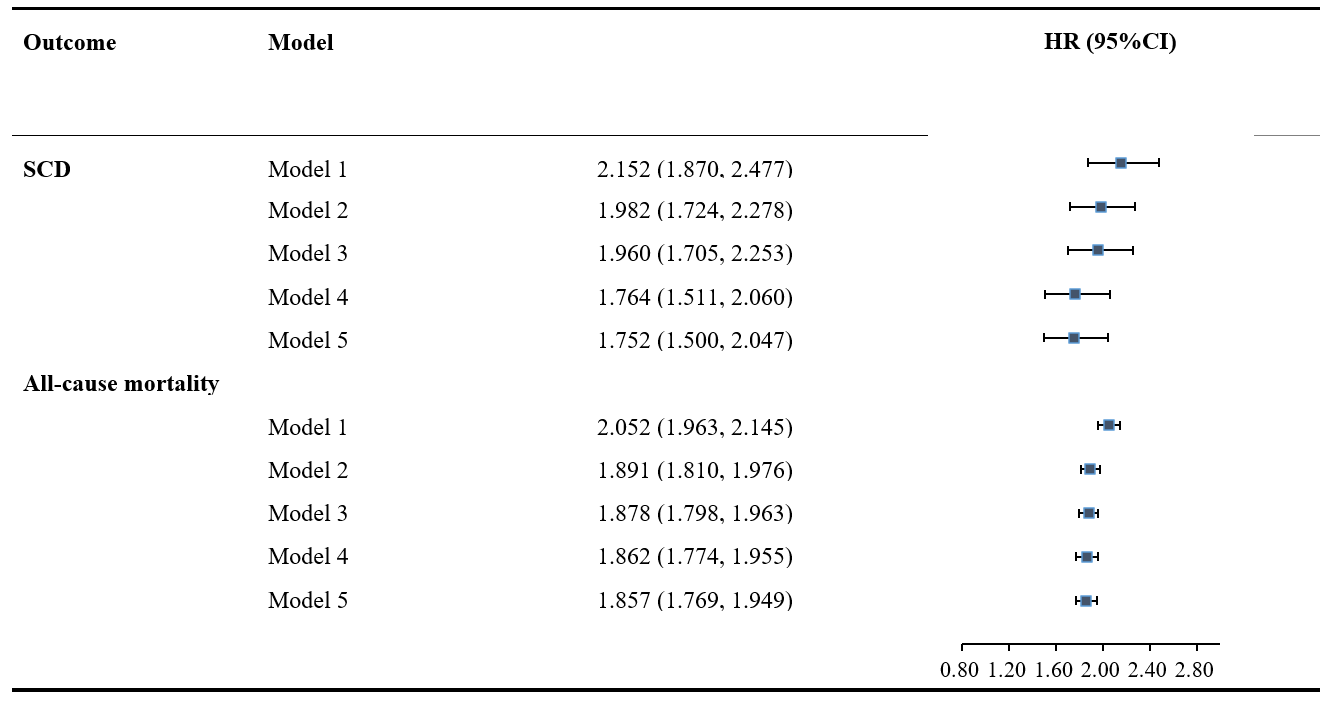


HR: hazard ratio. Model 1 unadjusted; model 2 adjusted for age and gender; model 3: model 2 plus BMI, physical activity, current smoking status, SBP and DBP; model 4: model 3 plus FBG, T, TG, BUN, TBIL and Scr; model 5: model 4 plus QTc prolongation and ST wave abnormality. Reference was the non-CMD group.

**Figure S4 Forest plots of the association of the number of cardiometabolic multimorbidity with sudden cardiac death and all-cause mortality in sensitivity analyses (excluding the deaths occurring in the first 2-year of follow-up)**
